# Evaluating Childhood Vaccination Coverage of NIP Vaccines: Coverage Survey versus Zhejiang Provincial Immunization Information System

**DOI:** 10.3390/ijerph14070758

**Published:** 2017-07-11

**Authors:** Yu Hu, Yaping Chen

**Affiliations:** Institute of Immunization and Prevention, Zhejiang Center for Disease Control and Prevention, Hangzhou 310000, China; zjmyscyp@163.com

**Keywords:** vaccination coverage, children, immunization information system, coverage survey, national immunization program, vaccine

## Abstract

Vaccination coverage in Zhejiang province, east China, is evaluated through repeated coverage surveys. The Zhejiang provincial immunization information system (ZJIIS) was established in 2004 with links to all immunization clinics. ZJIIS has become an alternative to quickly assess the vaccination coverage. To assess the current completeness and accuracy on the vaccination coverage derived from ZJIIS, we compared the estimates from ZJIIS with the estimates from the most recent provincial coverage survey in 2014, which combined interview data with verified data from ZJIIS. Of the enrolled 2772 children in the 2014 provincial survey, the proportions of children with vaccination cards and registered in ZJIIS were 94.0% and 87.4%, respectively. Coverage estimates from ZJIIS were systematically higher than the corresponding estimates obtained through the survey, with a mean difference of 4.5%. Of the vaccination doses registered in ZJIIS, 16.7% differed from the date recorded in the corresponding vaccination cards. Under-registration in ZJIIS significantly influenced the coverage estimates derived from ZJIIS. Therefore, periodic coverage surveys currently provide more complete and reliable results than the estimates based on ZJIIS alone. However, further improvement of completeness and accuracy of ZJIIS will likely allow more reliable and timely estimates in future.

## 1. Introduction

In China, recommendations on the immunization schedules of national immunization program (NIP) are made by Chinese advisory committee on immunization practices (CACIP), which is a group of medical and public health experts. According to the CACIP’s guidelines issued in 2008, each child should be protected against 12 infectious diseases by receiving at least 22 vaccinations in a timely manner before their seventh birthday (Table 1). Vaccinations included in the NIP are administrated in immunization clinics free of charge and are recorded in vaccination cards.

Accurate information on the vaccination status of target children is needed for program management and interventions. Vaccination coverage is the most frequently adopted indicator for evaluating the performance of immunization programs worldwide [1]. According to the guidelines by the World Health Organization (WHO) [2], vaccination coverage can be monitored through administrative data or periodically vaccination coverage surveys. In China, previous studies have identified delayed administration of specific vaccine doses as a potential barrier for the success of immunization program, despite the high coverage achieved [3,4,5]. Hence, monitoring the timeliness of vaccination helps verify that vaccine doses are administered properly to minimize the time that the child is at risk of infection.

With the increasing use of the immunization information system, the growing potential of central registers to create data in supporting the immunization program has been emphasized [6]. Also, using an immunization information system to evaluate the vaccination coverage can address the disadvantages of the coverage survey (i.e., larger resources on time, financial, and language barriers in the field survey). Zhejiang is a province located in east China, with 70 million inhabitants living in 11 municipalities. Zhejiang has an annual birth cohort of approximately 700,000 newborns. Since 2004, the Zhejiang provincial immunization information system (ZJIIS) has been established and been accessible for all immunization clinics [7]. ZJIIS contains a client application software deployed in immunization clinic and a central data base deployed in Zhejiang provincial center for disease control and prevention (CDC). The demographic information of children and vaccination information are collected, then transferred to the central database in real time. ZJIIS also has a web-based management system to assess the coverage and to better manage the immunization program.

We had attempted to use ZJIIS to evaluate the coverage of NIP vaccines and some self-paid vaccines (not included in NIP and voluntary, such as varicella vaccine) among the specific populations [7,8,9]. However, these studies had a common limitation in that the results were based on the immunization records of children registered in ZJIIS. It meant that only using the ZJIIS could not provide reliable estimates of coverage and it might be overestimated, because we did not know the proportion of children not registered in ZJIIS and the vaccination coverage of was lower for all vaccines among children not registered. In fact, many migrant children who are registered or receive the precedent vaccinations out of Zhejiang province may be missed to be registered in ZJIIS due to lacking of a unified national immunization register and the information exchange.

This study aimed to evaluate the degree of underestimation of vaccination coverage and timeliness derived from ZJIIS at provincial level, compared to the most recent vaccination coverage survey at a provincial level, implemented in 2014.

## 2. Methods

### 2.1. Design of the 2014 Coverage Survey

In 2014, 2772 children aged 24–35 months (born from 1 September 2011 to 31 August 2012), were recruited for participation in a vaccination coverage survey. Children were randomly selected from 66 towns, 6 towns for each municipality, through a household-based two-stage cluster design recommended by WHO [10].

The sample size was obtained based on the formula of $N=deff\times \frac{z_{\left(1-\alpha /2\right)}^{2}\;\times \;p\;\times \;\left(1-p\right)}{d^{2}}$. To reach the estimate of coverage at city level with a two-tailed α error of 5% and a permissible error (d) of 0.08, assuming the expected up-to-date fully immunization (UTDFI) coverage (*p*) at 70% and a design effect (*deff*) of 2, the minimum sample size required for each city was 252 eligible children, divided in 6 clusters (towns) of 42. The sampling procedures included four steps. First, six clusters for each city were selected from a list of all towns (with the population size) under the jurisdiction, on the basis of the probability proportional to population size. Second, one community was randomly selected from each cluster through the simple ballot from the list of all communities under the jurisdiction. Third, the index household was selected randomly from the list of all households in the selected community, by using the table of random numbers. Fourth, we selected the subsequent 41 households, by turning to the right while exiting the index household and visiting the adjacent one.

### 2.2. Data Collection

The 2014 coverage survey was based on the information gathered through a face to face interview with children’s parents, in addition with the consultation of ZJIIS. Information regarding the immunization history of children was first collected from vaccination cards through the interview, and then was further completed or validated through ZJIIS. Only documented doses until the day of interview (from vaccination cards or ZJIIS), were considered as administrated.

### 2.3. Outcome Measures and Data Analysis

We used Microsoft Excel 2013 for data analysis. The proportions of under-registration or missing vaccination cards were estimated by comparing the status of holding vaccination cards of individual child according to the survey with their respective status of registration in ZJIIS.

Given the efforts that were made to complete or validated through ZJIIS on the same day of the face to face interview, the survey data was considered as a proxy of the final true vaccination status of every child. The vaccination coverage was defined as the proportion of children having received the specific vaccinations, independent of their age at vaccination. The timeliness of vaccination was defined as the proportion of children having received the specific vaccinations within one day after birth for HBV1 and BCG or within one month of the recommended age for other vaccinations. The coverage and the timely administration of NIP vaccine doses were calculated.

The validity of data recorded in ZJIIS was evaluated by comparing the dates of administration with those recorded in the vaccination cards through survey, considering the latter as correct. This comparison was limited to vaccinations that were recorded in both vaccination cards and ZJIIS, because ZJIIS was used to complete the missing data in vaccination cards or to correct the possible transcription errors during the survey. The corresponding dose number was derived from vaccination cards for comparison of the administration dates, even if some vaccination doses might not have been registered in ZJIIS. For instance, if one individual record in ZJIIS contained three doses of DTP and if the first two doses corresponded to the dates transcribed from the vaccination card but the date of the third registered dose corresponded to the fourth dose recorded in the vaccination card, the third dose of DTP was considered as missing in ZJIIS.

### 2.4. Ethical Considerations

This study was approved by the ethical review board of Zhejiang provincial CDC (T-043-R). Written informed consent was obtained from a parent or a legal caregiver of each child enrolled in the 2014 survey, including the consent to consult ZJIIS to check or complete vaccination status.

## 3. Results

### 3.1. Source of Vaccination History

Of the enrolled 2772 children in the 2014 coverage survey of Zhejiang province, the proportions of children with vaccination cards and registered in ZJIIS were 94.0% and 87.4%, respectively. Totally, 2397 (86.5%) children had both vaccination card and registration in ZJIIS, and 142 (5.1%) children had neither vaccination card nor registration in ZJIIS, and 208 (7.5%) children had vaccination cards but did not register with the ZJIIS, and 25 (0.9%) children registered with the ZJIIS had missed their vaccination cards. There were 350 children who were registered in the ZJIIS, and the under-registration rate was 12.6% (Table 2).

### 3.2. Comparison of Vaccination Coverage

The coverage rates from ZJIIS were systematically higher than the corresponding estimates obtained through the survey. The mean difference was 4.5%, and the range of difference was from 3.0% to 6.6%. This difference seemed to increase by the dose number of multiple doses vaccines (i.e., DTP), because subsequent doses of these vaccines sometimes were unregistered in ZJIIS (Table 3).

### 3.3. Accuracy of ZJIIS

Among the 2397 children, there were 39,057 vaccination doses (95.8%) registered in ZJIIS and recorded in the corresponding vaccination cards meanwhile. Overall, 16.7% of the vaccination doses registered in ZJIIS differed from the date recorded in the corresponding vaccination cards. In general, the difference of the administration date between ZJIIS and survey was limited to less than one week (14.5%). A difference between 7 and 31 days was found in 1.8% doses and only very few cases (0.4%), the difference between two resources exceeded one month (Table 4). The difference was higher for vaccinations recommended in the second year of life, where the administration dates were equal in 78.6–80.4% of the relevant doses.

## 4. Discussion

Vaccination coverage and surveillance of vaccine preventable disease are two key measurements of immunization program. Accurate information on immunization status of target population is necessary both at individual and different administrative levels, to detect sub-populations or sub-geographic areas at high risk and adjust the immunization strategies [11]. At the individual level, using ZJIIS allows the providers to rapidly evaluate whether a child require a vaccination, and enables recall and reminder strategy, and facilitates opportunistic vaccination.

In this study, we assessed the registration rate, completeness, and accuracy of vaccination data in ZJIIS, by comparing with the results from the 2014 coverage survey. We found ZJIIS systematically overestimated the vaccination coverage compared with the 2014 survey results. Incomplete registration in ZJIIS significantly influenced on the vaccination coverage estimates derived from ZJIIS, and led to an overestimation of the coverage which had been demonstrated elsewhere [12,13]. Although all immunization clinics in Zhejiang province had used ZJIIS to register the children as well as record the vaccination information in their jurisdictions, an under-registration rate of 12.6% among the surveyed children was still observed in our study. The main reason for under-registration was immigration of children. Zhejiang is a socio-economically developed province and has attracted more than 20 million migrant people from other areas of China since 1990s. Consequently, there are almost 200 thousand migrant children for each birth cohort. These migrant children who lived in Zhejiang but are registered or receive the precedent vaccinations in their hometowns. On the other hand, some who are born in Zhejiang but living in other provinces for a period of time. Hence, these migrant children would be missed in the ZJIIS registry by immunization clinics. Furthermore, some migrant children with high frequency of immigration may live in Zhejiang province for a short period of time or receive only 1–2 vaccinations. Data of those children are asked to be recorded in ZJIIS by immunization clinics, but this is rarely done as some of the physicians think it is time-consuming and meaningless [7]. As of 2016, an incentive strategy that the vaccination providers will get a financial reimbursement from the local governments of 5 CNY per registration of a migrant child, was released and implemented [14]. We think that this strategy will encourage the physicians to register the migrant children and help to facilitate the retrieval of the immunization information administrated out of Zhejiang province. Another important reason lies in the fact that ZJIIS is currently limited to the immunization clinics in Zhejiang province. As we know, more than 20 provinces have established the immunization information systems in China, but a unified national immunization register or the standards of immunization information system has not been developed, as has been done in Canada [15]. Alfosni [16] had demonstrated the challenges that were faced when different immunization information systems are adopted at local level. There was no consensus on the minimal dataset collected and on the type of tool used for data management, which were both necessary for ensuring the quality and timely transmission of immunization data and availability of accurate estimates on vaccination coverage.

In addition to the incomplete registration, less than 1% of the immunization data was available in ZJIIS but the relevant vaccination cards were missing. It indicated that using an electronic based immunization information system with comprehensive access for all providers thus avoided data loss through missing vaccination records kept by parents. In this study, we found the increased trends of overestimation of coverage and inaccuracy of administration date obtained from ZJIIS during boosters or subsequent doses of multiple doses vaccines. The potential reason for this may be attributed to a decreased compliance by vaccination providers in making entries into the ZJIIS, possibly underestimating the importance of recording all vaccinations and not just those of infancy.

In this study, the differences in administration date were observed, for 16.7% of doses. Most of them deviated less than one week. It may be explained by the fact that all vaccination providers were required to record the intraday immunization information in both ZJIIS and paper archives before 2016. As such, some of the providers would choose to record the immunization information in paper archives as priority. We speculated that a considerable proportion of vaccination providers had a delay in recording the administrated vaccinations due to this reason, and the default entry date within the dropdown list is the current date of accessing the database. On the other hand, reliability of date recorded in the vaccination card would also be doubtful, as transcription errors might sometimes occur.

Although coverage estimates from surveys are often trusted more than the administrative estimates, survey studies are subject to other types of information bias, selection bias, and sampling error, as well as having high resource demands for time and money [17]. As such, using immunization information system like ZJIIS would be an alternative to monitor the vaccination coverage as it is often a quicker and cheaper way for immunization program managers to get more actionable information. Moreover, ZJIIS can be used to rapidly identify children with missed vaccinations and recall them for catch-up through telephone calls or text messages, as have been suggest and proved efficient in some other settings [18,19]. This contact information on immunization also needs to be updated and expanded on a regular basis.

There was a limitation in our study. Both the under-registration rate and the deviation in the date recorded in the ZJIIS versus the 2014 coverage survey showed an increasing trend for subsequent doses, but we could not explain this phenomenon using the available data, and we suggested that future research should be focused on identifying the specific reasons for this issue.

## 5. Conclusions

Accurate estimates on childhood vaccination coverage are necessary for formulating recommendations and interventions of the immunization program. Despite being staff intensive and expensive, the survey study provided more complete and reliable estimates than ZJIIS, in addition to a set of information on parents’ behavior on immunization and specific reasons for under-vaccination at the current time. However, population and big data based ZJIIS holds a great potential in evaluating the vaccination coverage. On the basis of improvement in ZJIIS, more accurate coverage estimates will likely be available in a timely manner than through traditional methods. Furthermore, ZJIIS can keep individual immunization information in a long time, which will help to make registration of vaccination in a life time more feasible.

## Figures and Tables

**Table 1:** Chinese childhood vaccination schedule up to six years of age.

Age	Vaccination
Birth	BCG + HBV1
1 month	HBV2
2 months	PV1
3 months	PV2 + DTP1
4 months	PV3 + DTP2
5 months	DTP3
6 months	HBV3 + MPV-a1
8 months	MR + JEV1
9 months	MPV-a2
18 months	MMR + DTP4 + HAV
2 years	JEV2
3 years	MPV-ac1
4 years	PV4
6 years	MPV-ac2 + DT

BCG: Bacillus Calmette–Guérin live attenuated vaccine; HBV: hepatitis B virus vaccine; PV: poliovirus vaccine; DTP: diphtheria–tetanus–pertussis combined vaccine; MPV-a: meningococcal polysaccharide vaccine type a; MR: measles–rubella combined live attenuated vaccine; JEV: Japanese encephalitis virus live attenuated vaccine; MMR: measles–mumps–rubella combined live attenuated vaccine; HAV: hepatitis A virus vaccine; MPV-ac: meningococcal polysaccharide vaccine types a and c; DT: diphtheria–tetanus combined vaccine.

**Table 2:** Source of obtained vaccination history of the 2772 children in the 2014 coverage survey, Zhejiang province.

Source of Vaccination History		Registered in ZJIIS (*n*, %)		Total
		Yes	No	
Holding vaccination card during the survey (*N*, %)	Yes	2397 (86.5)	208 (7.5)	2605 (94.0)
	No	25 (0.9)	142 (5.1)	167 (6.0)
Total		2422 (87.4)	350 (12.6)	2772 (100.0)

**Table 3:** Estimated vaccination coverage among children aged 24–35 months of age in Zhejiang province, based on the 2014 coverage survey and ZJIIS.

Vaccination	Estimate from Survey (%, *N* = 2772)			Estimate from ZJIIS (%, *N* = 2422)	Difference between Two Estimates (%)
	Vaccination Card	ZJIIS	Total		
BCG	91.6	0.8	92.3	97.3	5.0
HBV1	94.1	0.8	94.9	97.9	3.0
HBV2	93.1	0.8	93.9	97.6	3.7
HBV3	90.9	0.7	91.6	97.2	5.6
PV1	93.5	0.8	94.3	97.6	3.3
PV2	92.8	0.8	93.6	97.4	3.8
PV3	92.1	0.6	92.7	97.0	4.3
DTP1	93.0	0.8	93.7	97.4	3.7
DTP2	91.8	0.7	92.5	96.9	4.4
DTP3	91.5	0.6	92.1	96.7	4.6
DTP4	89.1	0.5	89.7	96.0	6.3
MR	90.8	0.7	91.5	96.7	5.2
MMR	92.0	0.6	92.7	96.4	3.7
HAV	89.0	0.5	89.4	96.0	6.6
JEV1	91.3	0.6	91.8	96.3	4.5
MPV-a1	91.2	0.7	91.9	96.7	4.8
MPV-a2	90.9	0.6	91.5	96.3	4.8

The vaccinations in gray shades were scheduled in the second year of life.

**Table 4:** Accuracy of administration dates in ZJIIS.

Vaccination	The Number of Doses Both Recorded in Vaccination Cards and Registered in ZJIIS	Equal Date (%)	Date Difference < 1 Week (%)	Date Difference 7–31 Days (%)	Date Difference > 1 Month (%)
BCG	2355	86.3	12.4	1.3	0.0
HBV1	2391	89.2	10.1	0.7	0.0
HBV2	2356	88.4	10.7	0.9	0.0
HBV3	2279	84.2	14.7	0.9	0.2
PV1	2353	85.7	13.1	1.2	0.0
PV2	2291	82.8	14.6	2.4	0.2
PV3	2175	80.9	15.0	3.6	0.5
DTP1	2369	84.6	13.8	1.6	0.0
DTP2	2304	83.2	14.7	2.0	0.1
DTP3	2268	81.2	16.2	2.2	0.4
DTP4	2204	78.6	18.1	2.1	1.2
MR	2319	84.6	13.6	1.5	0.3
MMR	2260	80.4	16.0	2.9	0.7
HAV	2238	80.2	15.5	3.1	1.2
JEV1	2318	84.8	13.3	1.5	0.4
MPV-a1	2327	83.9	14.4	1.5	0.2
MPV-a2	2250	81.6	15.6	2.2	0.6
Total	39,057	83.3	14.5	1.8	0.4

The vaccinations in gray shades were scheduled in the second year of life.
